# Vagus nerve stimulation for pediatric patients with intractable epilepsy between 3 and 6 years of age: study protocol for a double-blind, randomized control trial

**DOI:** 10.1186/s13063-018-3087-4

**Published:** 2019-01-14

**Authors:** Taoyun Ji, Zhao Yang, Qingzhu Liu, Jianxiang Liao, Fei Yin, Yanhui Chen, Liping Zou, Baomin Li, Yuxing Gao, Xiaomei Shu, Shaoping Huang, Feng Gao, Jianmin Liang, Su Fang Lin, Jing Peng, Shiwei Song, Jing Wang, Chao Che, Wenxiu Sun, Maoqiang Tian, Lin Yang, Yi Hua, Yunpeng Hao, Lixin Cai, Luming Li, Yuwu Jiang

**Affiliations:** 1 Division of Pediatric Neurology, Pediatrics Department, Peking University First Hospital, No.1 Xi’an Men Street, West District, Beijing, 100034 China; 2 Department of Pediatric Epilepsy Center, Peking University First Hospital, No.1 Xi’an Men Street, West District, Beijing, 100034 China; 3 National Engineering Laboratory for Neuromodulation, School of Aerospace Engineering, Tsinghua University, Beijing, China; 4 Department of Neurology, Shenzhen Children’s Hospital, Shenzhen, China; 5 Department of Pediatrics, Xiangya Hospital of Central South University, Changsha, Hunan China; 6 Hunan Intellectual and Developmental Disabilities Research Center of Children, Changsha, Hunan China; 7 Division of Pediatric Neurology, Pediatrics Department, Fujian Medical University Union Hospital, Fuzhou, China; 8 Department of Epilepsy Center, Fujian Medical University Union Hospital, Fuzhou, China; 9 Department of Pediatric, Chinese PLA General Hospital, Beijing, China; 10 Pediatics Department, Qilu Hospital of Shandong University, Jinan, Shandong China; 11 Division of Pediatrics Neurology, Provincial Hospital Affiliated to Shandong University, Jinan, China; 12 Department of Pediatrics, Affiliated Hospital of Zunyi Medical College, Zunyi, Guizhou China; 13 Department of Pediatrics, The Second Affiliated Hospital of Xi’an Jiaotong University, Xi’an, China; 14 Department of Neurology, The Children’s Hospital, ZheJiang University School of Medicine, Hangzhou, China; 15 Department of Pediatric Neurology, First Bethune Hospital, Jilin University, Changchun, China; 16 Research Center of Neuroscience, First Bethune Hospital, Jilin University, Changchun, China; 17 Department of Neurosurgery, Fujian Medical University Union Hospital, Fuzhou, China; 18 Man-Machine-Environment Engineering Institute, School of Aerospace Engineering, Tsinghua University, Room_204, North Part, Mengminwei Technology Building, Beijing, 100084 China; 19 Precision Medicine and Healthcare Research Center, Tsinghua-Berkeley Shenzhen Institute, Shenzhen, China; 20 Center of Epilepsy, Beijing Institute for Brain Disorders, Beijing, 100069 China

**Keywords:** Pediatric intractable epilepsy, Vagus nerve stimulation, Efficacy, Safety

## Abstract

**Background:**

Recent clinical observations have reported the potential benefit of vagus nerve stimulation (VNS) as an adjunctive therapy for pediatric epilepsy. Preliminary evidence suggests that VNS treatment is effective for seizure reduction and mental development in young participants between 3 and 6 years of age who suffer from intractable epilepsy. However, robust clinical evidence for quantifying the difference of the efficacy and safety of VNS treatment in this specific patient population has yet to be reported.

**Methods/design:**

A two-armed, multicenter, randomized, double-blind, prospective trial will be carried out to evaluate whether VNS is beneficial and safe for pediatric epilepsy. Pediatric participants aged between 3 to 6 years old with intractable epilepsy will be recruited and randomly assigned to experimental and control groups with a 1:1 allocation using a computer-generating randomization schedule. Before enrollment, informed consent will be signed by the parents of the participants and the study researchers. Participants in the experimental group will receive electrical stimulation over 24 weeks under standard stimulation parameters. Participants in the control group will not receive any stimulation during the 12 weeks of the double-blind period. The guardians of the participants are required to keep a detailed diary to record seizure activity. Outcome assessments including seizure frequency, Gesell Mental Developmental Scale scores, use of antiepileptic drugs and dosages, and adverse events will be collected at baseline, 6, 12, 18 and/or 24 weeks after electrical stimulation is initiated. The effects of treatment will be analyzed with time and treatment group comparisons.

**Discussion:**

This trial will evaluate quantitative differences in efficacy and safety with/without VNS treatment for pediatric participants aged between 3 to 6 years with intractable epilepsy and will explore whether the current age range of VNS therapy can be expanded.

**Trial registration:**

ClinicalTrials.gov, ID: NCT03062514, Registered on 23 February 2017.

**Electronic supplementary material:**

The online version of this article (10.1186/s13063-018-3087-4) contains supplementary material, which is available to authorized users.

## Background

Epilepsy is a brain disorder with symptomatic manifestation of uncontrolled seizures affecting patients of all ages [1]. Over 65 million people worldwide are estimated to have epilepsy, including approximately 10.5 million patients of which are children [1, 2]. Around one third of patients are resistant to antiepileptic drugs (AEDs) resulting in morbidity and high mortality, with a similar proportion being found in both adults and children [3]. Pediatric patients with drug-resistant epilepsy demonstrate age-related seizure expression but the reasons for such an occurrence are not fully understood [2]. Recurrent seizures in children have a high negative impact on physical growth, mental development, sleep and contribute to a heightened rate of behavior disturbances and psychiatric disorders (such as depression, anxiety, psychosis, suicide) later in life, bringing a heavy burden to families and care givers [2–9].

In 1997, the US Food and Drug Administration (FDA) approved VNS as an adjuvant treatment for patients with refractory epilepsy aged ≥ 12 years, who were poor candidates or had failed to gain curative effects by various therapeutic approaches such as ketogenic diet, resection surgery, and palliative surgery [10–13]. The commonly used VNS system (Cyberonics, Inc., Houston, TX, USA) was recently approved by the FDA (2017) for use in patients over 4 years of age who exhibit partial-onset seizures, who are refractory to antiepileptic medications based on retrospective analysis [14]. Indeed, many clinical studies suggest that VNS therapy is as effective in children as it is in adult patients [15–27]. A retrospective study using a large sample size in children (*N* = 347) has shown that VNS reduces the frequency of seizures and is well tolerated for over 2-years’ follow-up [26]. More specifically, 40–50% pediatric patients had reached > 50% reduction compared to baseline seizure frequency of the predominant seizure type. Several studies have found similar levels of efficacy for VNS treatment in pediatric patients with refractory epilepsy during 1–10 years of treatment [10, 15, 20–22, 24, 27–33]. Interestingly, age may be an important factor affecting the efficacy of VNS in children, as previous data have shown that younger participants demonstrate greater improvements following VNS intervention [26, 34]. In China, the domestic vagus nerve stimulator (G112, PINS Medical, Ltd., Beijing, China) has so far been implanted in 283 epileptic patients, with 32 participants being within the age range of 1–6 years. For these 32 pediatric participants, 8 participants showed adverse effects (25%, 8/32) including onset of coughing and notable voice change. However, all adverse reactions disappeared following changes to stimulation parameters. Furthermore, seizure frequency of 20 pediatric participants was reduced by > 50% (data not published).

Intractable epilepsy is associated with negative impact on development in children such as in cognition [6, 35], social behavior and maturation, academic achievements, language processing, and psychology, which causes a worsening of quality of life (QOL) [35–39]. Early and more effective seizure control is suggested to improve cognitive outcomes and QOL in children, which has been reported in several retrospective studies investigating the effects of epileptic surgery [40, 41]. It is well recognized that some AEDs often exert a negative impact on cognition and psychological development [4, 5], while VNS has not been associated with such adverse effects on cognitive developments. Indeed, most studies have observed stable cognitive function during VNS treatment, with some studies reporting mild positive effects on verbal performance, memory and/or mental state in children independent of seizure control [10, 11, 25, 35, 40].

According to the definition of drug-resistant epilepsy [42], Chinese clinical physicians always observe more than 2 years to before a diagnose of intractable epilepsy. Thus, the minimum age, 3 years old, in this trial could possibly be enough time to be examined and satisfy the inclusion criteria. In the largest sample size study of VNS in children, 46 participants (of 347,13.3%) aged < 6 years were enrolled but no specific outcomes were reported for this specific age range [26]. Lowering the age range for VNS treatment requires further clinical evidence. In this paper, we plan to conduct a multicenter, double-blind, prospective, randomized control study (VNS-PIE trial) in children with intractable epilepsy. The aim of this study is to quantitatively evaluate efficacy and safety of VNS treatment. The VNS-PIE trial is the first pilot study with children between 3 and 6 years of age with intractable epilepsy. Employing a randomized control trial (RCT) design, we assigned the first 12 weeks for double-blind comparisons from 2 weeks post VNS implantation. The latter 12 weeks were used to study the effects of treatment.

## Methods/design

### Type of trial

**Fig. 1:**
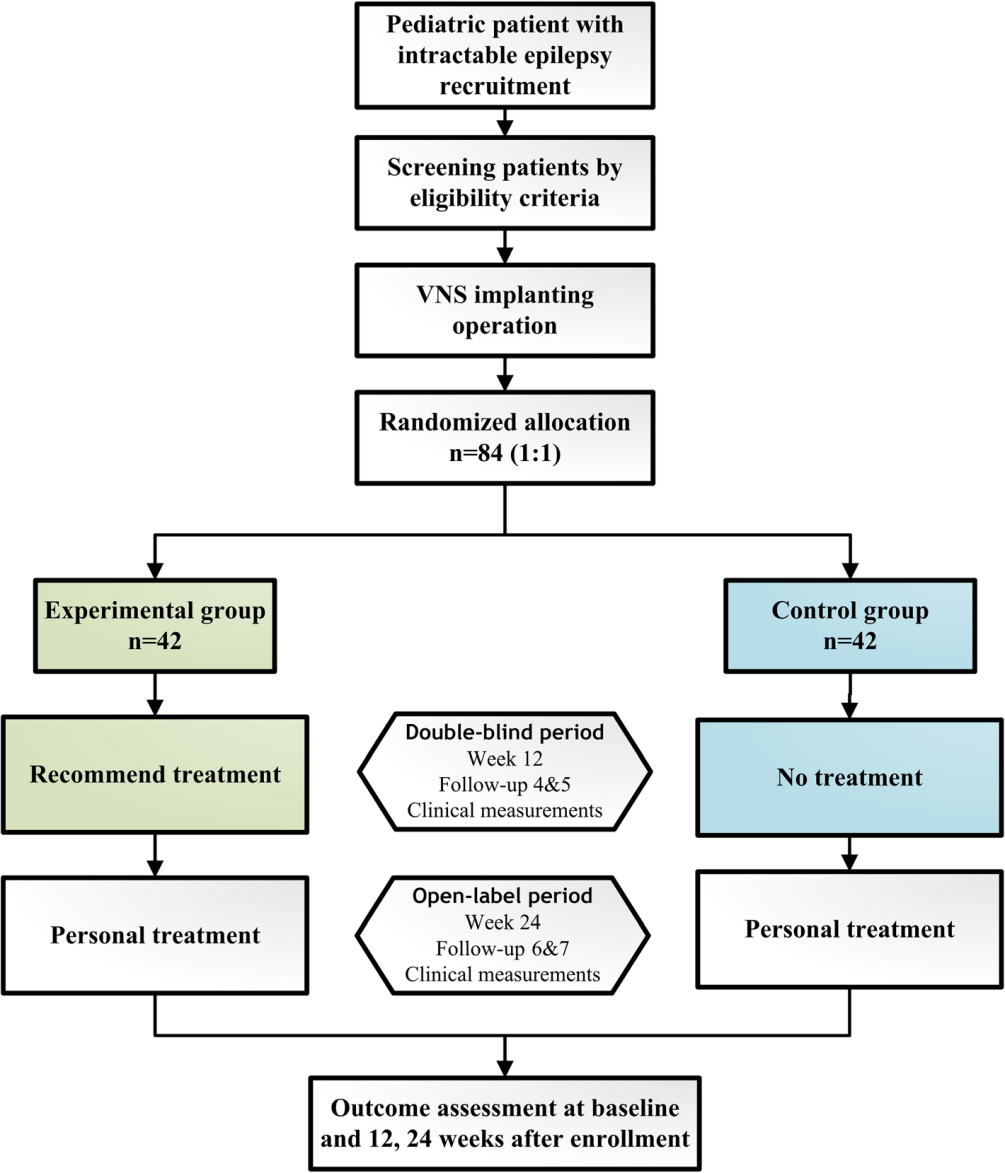
Flow chart of the VNS-PIE trial

**Fig. 2:**
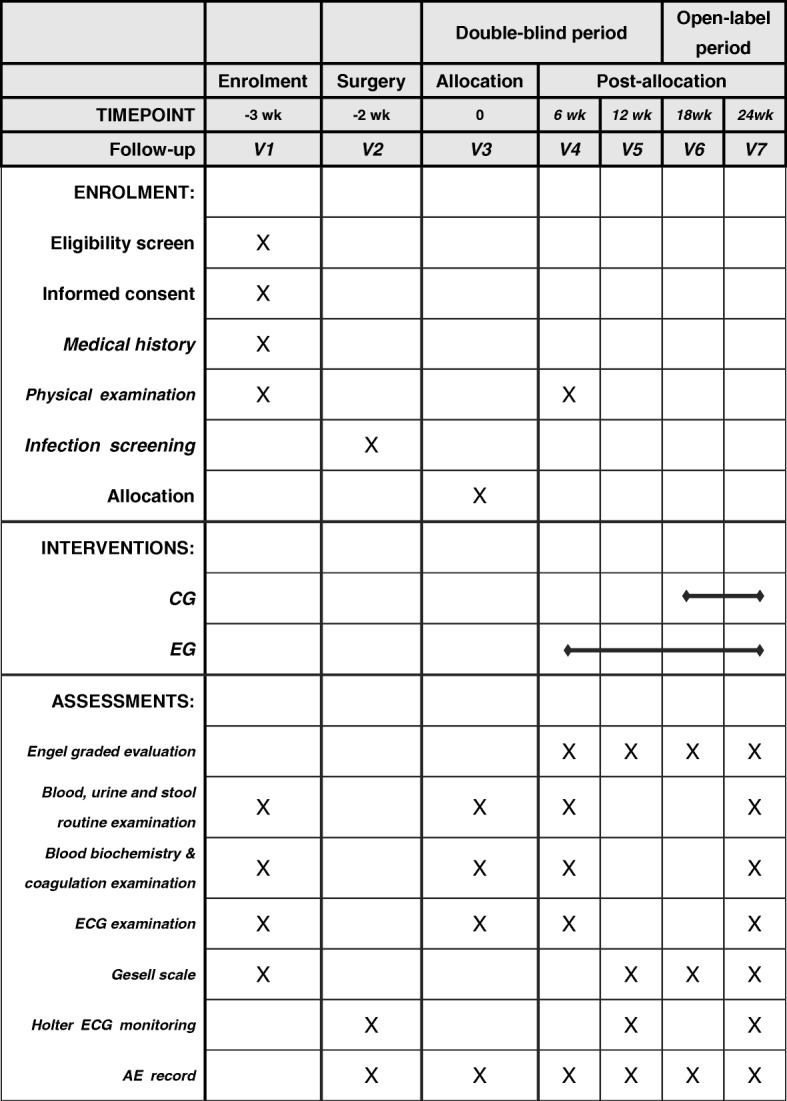
Standard Protocol Items: Recommendations for Interventional Trials (SPIRIT) Figure for the VNS-PIE trial

The VNS-PIE study is designed as a two-armed, randomized, double-blinded, multicenter prospective study. According to the trial flow chart (Fig. 1), pediatric participants with intractable epilepsy will be screened strictly based on the inclusion and exclusion criteria. A total of seven follow-up surveys are scheduled as shown in Fig. 2. For follow-ups V3–V7, participants will receive VNS treatment according to the group. The clinical measurements of the two groups will be examined based on Fig. 2. Prior to implantation, all participants will undergo baseline seizure evaluation, Gesell Mental Development Scale assessment and other clinical measures, including physical examination; blood, urine, stool routine analyses, blood biochemistry and coagulation, as well as electrocardiograph (ECG) examinations. At 2 weeks following operation, a total of 84 participants will be randomized (1:1) to one of two arms; the experimental group (EG) or the control group (CG) (*n* = 42 participants per arm). The participants in the EG will receive VNS therapy at 2 weeks post implantation until the end of the trial. The implantable pulse generator (IPG) of participants in the CG will not be switched on from the 2nd to the 14th weeks post operation. The outcomes will be calculated by blinded statisticians appointed by the Clinical Research Institute of Peking University following the end of the trial.

The VNS-PIE trial protocol was written in accordance with the Standard Protocol Items: Recommendations for Interventional Trials Statement (SPIRIT). A SPIRIT Checklist is included in Additional file 1. The trial will be carried out according to the principles of the Declaration of Helsinki (Edinburgh Version, 2000).

### Study setting

A total of 11 centers will participate in the VNS-PIE study. These include Peking University First Hospital (principal unit), Chinese PLA General Hospital, Shenzhen Children’s Hospital, Qilu Hospital of Shandong University, Shandong Province Hospital, the First Hospital of Jilin University, the Second Affiliated Hospital of Xi’an Jiaotong University, Xiangya Hospital Central South University, the Children’s Hospital Zhejiang University School of Medical, Fujian Medical University Union Hospital, and the Affiliated Hospital of Zunyi Medical College. The RCT design of this trial will allow for observations of safety and efficacy in the EG and CG, from the blinded and unblinded periods. The participants in the CG will not receive VNS treatment until 12 weeks after the EG begins receiving VNS treatment. One study reported that average seizure frequency reduction in very young group (< 8 years old) was nearly 50% after 3 and 6 months of VNS. However, The percentage of responders (seizure frequency reduction of 50% or more) increased with implantation time [2]. Thus, on the one hand, 24-week follow-up was considered adequate to observe the possible efficacy and safety difference between the EG and the CG and balance the blinding and unblinding stages. On the other hand, longer follow-up time for this RCT would be harder to be conducted considering participants’ compliance. Since protocol version 1.0 was first submitted to the Research Ethics Committee in September 2016, the Ethical Committee reviews have provided several rounds of review comments concerning to background, inclusion standard, comparator, clinical assessments, exit criteria, follow-up frequency, remedial measures, and other details. And the protocol was updated to version 3.0 in November 2016. In July 2017, all researchers and the sponsor held a meeting to discuss further amendments to the protocol with the mental development scale mainly changed. Protocol version 3.0 was finally updated to 3.1. The final version has been approved by the Clinical Trial Ethics Committee of Peking University First Hospital (Protocol number: G112 L31101; date: 31 July 2017) and registered at ClinicalTrials.gov protocol system (Clinical Trials Identifier: NCT03062514).

### Recruitment of participants

The target population for the VNS-PIE study is children aged between 3 to 6 years old who do not have any history of a vagus nerve lesion. Participants will be recruited by Internet advertisement and directly through the hospitals.

### Inclusion criteria

Inclusion criteria are shown in Table 1.

**Table 1:** Inclusion criteria of the VNS-PIE trial

Inclusion criteria	
1. Age 3–6	
2. At least 6 seizures per month	
3. Refractory epilepsy	
4. In good health except for epilepsy	
5. Family members of participants can understand the method and sign the informed consent	
6. Participants with good compliance and can complete post-operative follow-up	

### Exclusion criteria

Exclusion criteria are shown in Table 2.

**Table 2:** Exclusion criteria of the VNS-PIE trial

Exclusion criteria	
1. Results of magnetic resonance imaging (MRI) show that epilepsy is caused by intracranial space-occupying lesions	
2. Tumor, cardiopulmonary anomaly, heart failure, progressive neurological diseases, asthma, mental disease, peptic ulcer, diabetes, poor health, and other contraindications to surgery	
3. Vagus nerve lesion or damage	
4. Cannot write the epilepsy diary	
5. Participating in another clinical trial	
6. Cannot complete the operation	
7. Cannot complete the post-operative follow-up	
8. Cannot complete the programming	

### Sample size

We adopted a pilot study design to evaluate efficacy and to primarily quantify the frequency of seizure reduction between the EG and the CG at 12 weeks’ follow-up after VNS treatment. It has been estimated that the variation of seizure frequency in the EG (VNS + AEDs) will be conservatively higher (25%) than the CG (AEDs only). The standard deviation of the two groups is set at 35%. Therefore, the sample size calculation formula is:

1 $$ n=\frac{2{\left({\mu}_{\alpha /2}+{\mu}_{1-\beta}\right)}^2{\sigma}^2}{{\left({x}_T-{x}_C\right)}^2}, $$

where *x*_*T*_ is the expected curative effect of the EG, *x*_*C*_ is the expected curative effect of the CG, *σ* is the standard deviation of the two groups (35%), *μ* is the quantile of the standard normal distribution, *α* is the first-class error level for statistical test (0.025, here for unilateral test; 0.05 for bilateral test if need), and *β* is the second-class error level for statistical test (0.2, here). The calculated sample size per group through this calculation is 38. Considering that the maximum possible loss rate is 10%, the final sample size is 42 pairs (*N* = 84) [43–45] .

### Screening and enrollment

Research staff will check the medical history of participants (mainly the seizure and treatment history), perform a detailed physical examination and assess the participant’s auxiliary status (brain magnetic resonance imaging (MRI), electroencephalogram (EEG)) for eligibility. Once eligibility is confirmed, parents of participants are required to provide informed consent to researchers that specifies the possible risks and benefits of this trial. Researchers and parents should both sign their own names and date in handwritten form on the informed consent in duplicate copies. One copy will be given to the parents. In cases where the participant is not able to provide consent at the time of screening, the preoperative clinical measurements will not be arranged until the participants and their parents are able to give consent. Participants are numbered according to the registration sequence. Once participants are enrolled into the study, parents are required to record and keep daily diaries for occurrence of epileptic seizures.

### Randomization

The study participants will be randomly assigned to either the EG or the CG at a ratio of 1:1 at 2 weeks post surgery, according to the “random number table” generated by the BlueBALLANCE clinical trial central randomization system of the third party (Blue Balloons, Ltd., Beijing, China). There are two kinds of balancing factors: intra-block balance and systematic stochastic balance. Firstly, based on SAS software, each block has four ordered participants’ random numbers, randomly generated (two EG and two CG) to satisfy intra-block balance. Secondly, each time a participant registration number is entered into the BlueBALLANCE clinical trial central randomization system, a block placeholder is generated automatically on the stratification factor: medical center to which the participant belongs. Following the rules of the competitive block, if the current placeholder belongs to a block that has been occupied and has spare random number(s), the next spare random number in this occupied block is sequentially taken and assigned to the participant. Otherwise, a new block is occupied and the first random number inside this block is given to the participant. This operation ensures the random balance of the system. Each center designates a unique researcher to have a random system account and password. When a participant in one center needs their random number, the appointed researcher in this center enters the patient’s trial number into the system and obtains the unique random number of this participant from the computer operations, which then assigns the participant to the EG or the CG.

### Blinding

The study will be carried out under double-blind conditions with both investigator and participants (and their parents) being blind to the treatment given. Several approaches will be adopted to ensure that the blinded conditions of the clinical trial remain for assessing test treatment. i.e., stimulation intervention [46]. Researchers involved in this study will also be divided into two groups: blind and unblind. Researchers assigned to modulate the stimulation parameters and operate the randomized system will be unblinded. Other researchers, especially the evaluators, will be blind to treatment conditions until the end of the open-label period. Researchers blind to conditions will be trained to obey the experimental rules and not enquire into the randomized results. Normally, at 14 weeks’ follow-up after implantation surgery, participants in both the EG and the CG should be unblinded and the designated researcher will inform the random results to the participants and other researchers. During the double-blind stage, if one of two following specific circumstances occurs, participants can be unblinded. The first type is that there is an unacceptable deterioration and/or security issues that jeopardize the participant, especially in the CG. The unblinding process must be conducted after informing the sponsor or the sponsor’s authorized representative, with the consent of the participant, researchers, and sponsor. Stimulation parameters may be adjusted to a more reasonable treatment intensity by the researcher. As for participants in the CG, receiving stimulation during the double-blind stage means completely unblinding. The second circumstance is that the participant decides to withdraw from the trial during blinding and, therefore, the unblinded process will be executed immediately by the researcher. The time of unblinding, reasons for executing this process and operations need to be documented in hospital medical records and CRFs (case report forms) by the recording researcher. Accidental unblinding of participants also need to continue with follow-up observations. In such cases, their efficacy outcome after unblinding will not be included into the analysis of this trial. However, their safety outcome will still be included.

### Intervention phase

Following baseline and preoperative assessments, researchers will confirm the results collected within a normal clinical range and clinical significance. All participants will receive surgery for implantation of the VNS system (G112, PINS Medical, Ltd., Beijing, China). Participants will be observed for at least 2 days after surgery before being hospital discharged. For the first 12 weeks of VNS treatment, the recommended stimulation parameters for the EG will be as follows: frequency = 20–30 Hz; duration = 250–500 μs; stimulation cycle = 30 s; rest cycle = 5 min, electrical intensity = 0–1.5 mA and no magnetic stimulation. In the CG, no stimulation will be provided during the first 12 weeks. For the latter 12 weeks of treatment, personalized optimal stimulation parameters will be employed in the two groups. The pursuits of personal parameter adjustment are the alleviation of the seizures as much as possible, as well as nonoccurrence of adverse events (AEs) of the participants. The prescribed AEDs are not expected to be modified during this trial. Researchers may modulate the electrical intensity in gradual incremental steps of 0.1–0.5 mA for each parameter. If participants show symptoms of maladjustment at higher parameters, reduction will be recommended based on the researcher’s professional advice. For changes of any kind to the dose or type of AED, researchers are required to keep detailed records.

### Outcome measures

#### Efficacy outcomes

Following 12 weeks of VNS treatment, seizure frequency will be compared between the EG and the CG. This will be calculated as the primary measurement according to participant diaries and CRFs. Seizure outcomes will be expressed with a modified Engel scale [47].

For secondary outcomes of efficacy, change in seizure frequency, the modified Engel scale description, number of antiepileptic drugs used and Gesell Mental Development Scale scores will also be calculated and compared to baseline across the various time points of 6, 12, 18, and 24 weeks.

#### Safety outcomes

The occurrence rate of AEs/SAEs (serious adverse events) at distinct stages during the perioperative, double-blind, and open-label period will be used to evaluate the safety of treatment. If an adverse reaction occurs, the researcher will decide to take the necessary measures based on the health status of the participant. If a SAE occurs, the clinician will immediately turn off the IPG and assess the participant’s vital signs before providing appropriate symptomatic treatment. These events will be reported to the trial manager within 24 h. In extreme cases, the VNS system may be removed and the participant will be given post-trial care.

### Quality control and trial monitoring

Prior to the initiation of this clinical study, the primary investigator has formulated several documents including an investigator’s brochure, standard operating procedures (SOP), and a detailed research plan. All the staff involved in this clinical trial have participated in special training, including sessions on enrolling participants, completing the CRF and using the VNS system. To help avoid subjective differences in the grading between experimenters, researchers who are due to carry out the Gesell Mental Development Scale assessment have passed the consistency training course and been awarded graduation certificated by Peking University First Hospital.

In the VNS-PIE trial, clinical assessments, intervention parameters, and AE/SAE cases are documented on the CRFs. Each center has authorized researchers to finish data entry on the CRFs. If documented data are doubted, the major investigator gives clinical judgment and reasons for accepting or rejecting data. The researchers then make corresponding records or modifications on the CRFs. All the data in this trial will be kept in a special document cabinet by authorized researchers in each hospital. The files include the raw records, CRFs, and electronic documents. These data will be removed from hospitals only after the sponsor informs the clinical trial facility and researchers that it no longer needs the data. Before this, no data will be removed.

Data monitors, who are professional certificated clinical research associates (CRAs) from the sponsor (PINS Medical, Ltd., Beijing, China), will conduct monthly checks on contents of CRFs and the status of trial completion of each participation. Such reports will contain trial progress, discovered violation issues, historical records of issues resolved and revised, and the inspection results the center’s quality control organization. Each center’s quality control organization, independent from investigators and the sponsor, should inspect the trial’s quality and data management according to the trial plan and schedule (a minimum of three times). This includes, but is not limited to: when the first case is enrolled, when half of the enrollments have been completed and at the end of the trial.

Participants who do not complete the study or withdraw at any time in the trial will be noted. Reasons for withdrawal so will be fully documented. The trial sponsor will have full access to all datasets of the trial.

The final report will follow the Consolidated Standards of Reporting Trials (CONSORT) extension guidelines for non-pharmaceutical interventions. In order to minimize 6-month attrition, the first step will be to enroll participants whose guardians are aware of the beneficial effects of VNS therapy and who can understand the mechanisms of VNS. Secondly, in the consent form, the participant and their guardian will be informed that the participants can receive physician consultations throughout the whole study period. Moreover, participants will receive physical examination (Gesell Mental Development Scale), the VNS system and modulation of parameters free of charge if they complete the study. The physician will remain in contact for the participants’ medical needs throughout the study.

### Guidelines for stopping treatment

The trial may be ended prematurely by the Ethical Committee, medical supervision department and the sponsor if there are concerns of safety, or if there is evidence of harm in the study. The treatment in the trial can be interdictory when the researchers identify a SAE or if side effects occur under the regular increase of electrical stimulation parameters.

### Statistical analysis

To compare treatment efficacy and safety parameters between the EG and the CG, as well as within-group comparisons to baseline, data will be analyzed with *t* tests and *χ*^2^ tests for continuous variables and categorical variables, respectively. The Kolmogorov-Smirnov test or parametric tests will be used for analysis of data with normal distributions. Non-parametric data will be analyzed by a rank-sum test. The factors that impact curative effects will be analyzed with a multinomial logistic regression method. A *p* value < 0.05 will be considered as statistically significant. All analyses will be conducted using SAS 9.4 or higher version software.

### Confidentiality

All data generated in this study will be treated as confidential information belonging to the sponsor. With the exception of Chinese Food and Drug Administration (CFDA) procedures and compliance to relevant laws, researchers will be information confidential to any third parties and use such information only to the extent agreed, unless written consent has been granted.

The investigators must ensure that the privacy of the participant is maintained during and after the whole trial. Among all submissions, the identity of the potential and actual participant can only be determined by the abbreviation of their name and registration number.

## Discussion

The currently proposed, randomized controlled, double-blind clinical trial aims to quantify the efficacy and safety of VNS treatment in drug-resistant epileptic patients between 3 and 6 years of age. The results of this study may extend the age range of VNS therapy for clinical use.

Frequency and severity of seizures are the main parameters that are measured to illustrate the efficacy of VNS therapy in participants. Therefore, parents are required to document the observed frequency and average duration of daily seizures during this trial.

Early studies have demonstrated that children and adults have a similar extent of seizure reduction after VNS treatment following several months [15, 48, 49]. Orosz and colleagues have previously assessed change in seizure frequency following adjunctive VNS therapy in 347 children with drug-resistant epilepsy, with patients aged between 6 months to 17.9 years at the time of VNS implantation [26]. Data from this study showed that seizure frequency was reduced (32.5, 37.6 and 43.8%; at 6, 12, and 24 months, respectively) over a 2-year follow-up period. However, the findings of this study are limited in certain respects, such as lack of subgroup analysis based on age, which would be useful for separating patients on different stages of physiological development. While the number of participants aged < 6 years old in Orosz’s study was 46, the efficacy of VNS in patients was not released. It is important to note that age may be an important factor for the efficacy of VNS. This is indicated by a higher reduction of seizures in younger patients (< 12 years at implantation) than older patients (> 12 years at implantation), with the most beneficial patient group being children aged 0–6 years [19]. However, not all studies to date have shown differences in the efficacy of VNS between age groups [18, 22, 24, 28]. Interestingly, the age of seizure onset may be relevant to the clinical response. The earlier the age of seizure onset, the worse the VNS response may be because of the cumulative damage of epilepsy itself [27]. Therefore, implantation of VNS in children may be an effective means of controlling seizures at an early stage. Furthermore, VNS may reduce the severity of seizures in children with refractory epilepsy based on NHS3 scale measurements (baseline 9.5; endpoint:8.3; *p* < 0.001) [50–52]. Currently, in China, the CFDA approves VNS therapy in patients between the age of 6–60 years old. Few studies have been conducted in pediatric patients of an age range of 3–6 years old, which is an important period for growth and development in children [53]. Moreover, there are many types of epileptic syndromes in childhood according to classifications based on the age of seizure onset [53]. Interestingly, VNS treatment has shown a more curative response in certain epileptic syndromes (Dravet syndrome or Lennox-Gastaut syndrome) [26]. Considering this, we note that there is a need for a similar distribution of disease characteristics between the CG and the EG for this study.

Previous studies exploring VNS have reported stable outcomes or improvements in (subscales of) QOL [17, 33, 54, 55]. However, the QOL scale is not applicable to patients of 3–6 years of age. In comparison to commonly used drug treatments (such as phenobarbital, phenytoin, carbamazepine, and valproate) [56], VNS has no negative effects on cognitive and social ability. Participants with VNS treatment have demonstrated mood, attention, language, and memory improvements [25, 57].

Childhood development from birth to the age of 6 years old is essential for gross motor, fine motor, language, cognition, and social/emotional learning [58]. With the importance of measuring developments in a specific age range in children, the Gesell Mental Development Scale has been chosen for this trial [59–62]. This is because it is designed to provide relevance to the mental development in children aged between 3 and 6 years old [60, 62].

The adverse reactions of VNS treatment are mainly (80%) caused by the transient response induced by electrical stimulation. These AEs include voice change, dysphagia, and coughing, all of which are commonly alleviated following several hours of stimulation [15, 20, 63, 64]. More rare complications caused by VNS therapy includes respiratory sinus arrhythmia, which can reduce oxygen transport to brain tissue, aggravate the brain tissue injury of epileptic patients [30, 65]. During VNS treatment, sleep apnea can occur infrequently, but is especially dangerous for patients with obstructive breathing [66]. Equipment-related complications, electrode fracture (3%) or IPG malfunction (4%, such as power depletion, accidental power-off, too large/low impedance and unprogrammed electrical stimulation) [32], may also arise in patients. For younger patients, adolescent body growth and development may cause electrode fracture, which is the main complication that has been reported in previous studies [32, 67]. Complications associated with the VNS implantation operation are infrequent, with the most common issue being wound infection after surgery. The superficial infection can be controlled by antibiotics, while severe infections may require removal of the vagus nerve stimulator [27, 67, 68]. Stimulation, equipment and surgery-related AEs will be recorded on CRFs in order to assess the safety of VNS treatment for pediatric epilepsy.

It is important to note that this study has several potential limitations. Firstly, all the outcome measures of efficacy are self-assessments taken from a seizure diary. While objective measures of seizures can be made from an epilepsy-recording bracelet, this will not be used in this study because of the age of the patients and specificity of equipment to seizures (i.e., generalized tonic-clonic seizures) [69]. Here we will utilize daily diaries to assess changes in seizure frequency and severity, which have been a common method in RCTs for epilepsy. Another potential limitation of this clinical trial is the time duration of 24 weeks for follow-up. We note that longer follow-ups for assessing chronic VNS treatment over 1–2 years are valuable [27, 70].

## Trial status

Enrollment of participants into this study started in March 2017 and was completed in May 2018. Target enrollment for this study is 84 participants.

## Additional file

Additional file 1: Standard Protocol Items: Recommendations for Interventional Trials (SPIRIT) 2013 Checklist: recommended items to address in this clinical trial protocol and related documents. It is strongly recommended that this checklist is read in conjunction with the SPIRIT 2013 Explanation and Elaboration for important clarification on the items. Amendments to the protocol should be tracked and dated. The SPIRIT Checklist is copyrighted by the SPIRIT Group under the Creative Commons “Attribution-NonCommercialNoDerivs 3.0 Unported ” license. (DOC 124 kb)

## Abbreviations

AE: Adverse event
AED: Antiepileptic drugs
CFDA: Chinese Food and Drug Administration
CG: Control group
CRA: Clinical research associate
CRF: Case report form
ECG: Electrocardiograph
EEG: Electroencephalograph
EG: Experimental group
FDA: Food and Drug Administration
IPG: Implantable pulse generator
MRI: Magnetic resonance imaging
QOL: Quality of life
SAE: Serious adverse event
SOP: Standard operating procedure
VNS: Vagus nerve stimulation
